# Carbon Footprint Assessment of Dairy Milk and Grana Padano PDO Cheese and Improvement Scenarios: A Case Study in the Po Valley (Italy)

**DOI:** 10.3390/ani15060811

**Published:** 2025-03-12

**Authors:** Giulia Ferronato, Noemi Tobanelli, Paolo Bani, Luca Cattaneo

**Affiliations:** 1 Department of Civil Engineering, Architecture, Environment, Land Planning and Mathematics (DICATAM), Università degli Studi di Brescia, Via Branze, 43, 25123 Brescia, Italy; noemi.tobanelli@unibs.it; 2 Department of Animal Science, Food and Nutrition (DIANA), Faculty of Agricultural, Food and Environmental Sciences, Università Cattolica del Sacro Cuore, 29122 Piacenza, Italy; paolo.bani@unicatt.it (P.B.); luca.cattaneo@unicatt.it (L.C.)

**Keywords:** cheese, global warming potential, dairy, milk

## Abstract

The significance of agriculture, particularly dairy farming, in the global food production landscape has been ascertained. Farm efficiency affects how much the agri-food sector, and the dairy industry in particular, contributes to economic and environmental sustainability. This study employs an LCA approach to evaluate the carbon footprint (CF) of Grana Padano PDO cheese production in a dairy plant, analyzing 19 farms supplying milk to the cheese factory. The results showed that milk production is the primary contributor to CF, with enteric methane emissions (34%), feed production and purchases (36%), and manure management (24%) as the main drivers. The CF of milk ranged from 0.95 to 2.14 kg CO_2_eq/kg Fat and Protein Corrected Milk, while Grana Padano PDO cheese (9 months ripening) ranged from 16.96 to 23.07 kg CO_2_eq/kg. An increase in milk yield and feed efficiency resulted in a reduction in CF per kilogram of cheese. Furthermore, the protein and casein content influenced both cheese yield and environmental performance. This study highlights trade-offs between productivity, product quality, and sustainability, emphasizing the need for tailored mitigation strategies within PDO regulation.

## Simple Summary

This study examines the environmental performance of dairy farms in the Po Valley involved in the production of PDO Grana Padano and identifies the key factors influencing its sustainability. The results highlight the crucial role of high-quality feeding strategies in improving milk yield and reducing enteric methane emissions, nutrient excretion and manure emissions. Increasing milk yield—when combined with an appropriate nutritional balance—leads to a reduction in methane emissions per unit of milk and cheese, highlighting the strong link between productivity and environmental impact. The study also identifies a trade-off between optimizing milk yield and maintaining milk quality, particularly in terms of fat and casein content, which are essential for cheese production. This balance is crucial to ensure both economic viability and reduced environmental impact. Although focused on a specific region, these findings provide useful insights into strategies for reducing the carbon footprint of dairy farms. Future research should further investigate the role of different crop and livestock management practices, as well as the application of Nutritional LCA to improve the sustainability of dairy and cheese production.

## 1. Introduction

The agri-food sector has a pivotal role in global economic growth, food security, and rural development, besides supporting community livelihoods and preserving cultural heritage [1,2]. It is essential to acknowledge the significance of environmental sustainability in ensuring the sector’s long-term resilience and efficient use of resources. Livestock sector contributes 14.5% of total global anthropogenic greenhouse gas (GHG) emissions, with the largest overall contributors being beef and dairy cattle, which account for 65% of total livestock GHG [3,4]. In particular, dairy systems contribute to environmental impacts through both direct emissions (methane and nitrous oxide) and indirect impacts linked to feed purchasing and energy-intensive processing [5].

The contribution of the agri-food sector, and more specifically the dairy industry, to both economic and environmental sustainability is influenced by farm efficiency. This can be defined as the ability to convert inputs such as feed and resources into nutrient-rich outputs like milk, proteins, and fats [6,7]. Efficient dairy production is characterized by the maximization of nutrient utilization, ensuring that a greater proportion of ingested nutrients is converted into high-quality milk components, thereby reducing losses through enteric methane emissions, feces, and urine excretion [8]. Conversely, low efficiency is associated with nutrient losses, such as nitrogen as well as phosphorous, potassium and carbon in manure that leads to further emissions in air, soil, or water [9,10,11,12]. These inefficiencies not only contribute to environmental burdens but also reduce farm profitability, as resources are wasted rather than being used productively. Consequently, enhancing efficiency in dairy systems emerges as a pivotal strategy for mitigating environmental impacts while concurrently enhancing economic performance and resource sustainability [13,14,15].

The quality of milk is of pivotal significance for both human health and the optimal transformation into dairy products. At the farm level, the assessment of milk quality is primarily based on hygienic safety and the absence of undesirable compounds that could pose health risks or compromise its technological properties, such as antibiotics, harmful bacteria, or contaminants. Milk composition, particularly its protein and fat content, is a key determinant of its economic and technological value, influencing characteristics such as cheese yield, coagulation properties, and sensory attributes. In several dairy industries, farmers receive payments based on the quality of their milk, with bonuses or penalties allocated for traits such as total protein and casein concentration, given their direct impact on cheesemaking processes [16,17].

In the context of an increasing focus on sustainable food production, the integration of nutritional value into environmental assessments has emerged as a strategy for evaluating dairy sector efficiency. The nutrient-focused Life Cycle Assessment (nLCA) approach, recently endorsed by the Food and Agriculture Organization (FAO), provides a comprehensive framework for linking milk’s environmental footprint to its nutritional contribution. This method offers a more holistic assessment of dairy sustainability, ensuring that environmental impact evaluations account for the nutritional quality of milk rather than solely production volume [16,18,19].

In the context of environmental challenges, Protected Designation of Origin (PDO) dairy products, such as Grana Padano, have a pivotal role in the preservation of regional traditions, the assurance of product quality, and the promotion of rural economic development. The Grana Padano PDO supply chain, which is concentrated in the Po Valley (Italy), adheres to strict production regulations that define feeding strategies, milk composition, and cheese-making processes [20]. While these specifications safeguard product authenticity, they also impose constraints on environmental optimization, as certain mitigation strategies may conflict with PDO regulations. Consequently, it is essential to assess the environmental impact of Grana Padano production within its regulatory framework, identifying tailored mitigation strategies, especially with regard to feeding strategy, that enhance sustainability while maintaining product quality [21,22,23,24,25,26].

The present study aimed to evaluate the carbon footprint (CF) associated with the production of cow’s milk and dairy products at the farm gate, as well as Grana Padano PDO cheese at the factory gate. Additionally, the study examined CF variability and its key influencing factors within a relatively homogeneous sample, where the limited variation in environmental conditions—such as soil characteristics, climate, and local farming practices—allowed for a more precise assessment of production-related impacts. To this end, 19 dairy farms in the Po Valley (Piacenza province, Italy) were analyzed, and potential improvement scenarios were explored to identify the most effective mitigation strategies.

## 2. Materials and Methods

### 2.1. System Description

The raw milk used to produce Grana Padano PDO cheese must satisfy specific criteria. The production of this cheese is concentrated in a number of Italian regions, including Lombardy, Piedmont, Veneto and Trentino-Alto Adige, as well as a portion of Emilia-Romagna, specifically the Piacenza area. Firstly, it must be sourced from cows milked twice daily on dairy farms located within the designated production area [27]. Secondly, the cows’ diet must consist primarily of fresh or preserved forages (hay or silage) and concentrates, with the forage-to-concentrate ratio on a dry matter (DM) basis not exceeding 1 in the daily ration. Furthermore, at least 50% of the dry matter in the daily ration must be produced within the designated production area. The milk must not be subjected to temperatures below 8 °C, whether on the farm or during transportation to the cheese factory. The milk is subjected to partial skimming through natural creaming, whether derived from a single milking session or a combination of two. The use of lysozyme is permitted, with a maximum limit of 25 g per ton of milk. Grana Padano PDO labeling is authorized only after a minimum aging period of nine months, provided the product passes the official quality control procedures [20].

The sample analyzed consists of one dairy factory and its 19 members conferring farms located in the Piacenza province (Emilia Romagna region, Italy).

### 2.2. Life Cycle Assessment

The LCA methodology was performed in accordance with the ISO 14040 and 14044 [28,29] guidelines, as well as the ISO 14067 standard [30] for determining the carbon footprint (CF). Furthermore, the IDF guidelines for the dairy sector were taken into consideration [31].

### 2.3. Goal and Scope

The objective of the present study was to evaluate the carbon footprint of milk production in the Po Valley region through the utilization of a Life Cycle Assessment (LCA) approach. The study’s objectives were to identify the primary stages and processes contributing to the carbon footprint of raw milk at the farm gate and of Grana Padano PDO cheese ripened for 9 months at the cheese factory gate.

### 2.4. Functional Unit

Three functional units (FU) were considered. At the farm gate, the FUs were the 1 kg of Fat Protein Corrected Milk (FPCM) as suggested by FIL-IDF (Equation (1)) [31] and 1 kg of useful cheesemaking material (fat and protein).

FPCM (kg) = Milk yield (kg) × [0.1226 × Fat% + 0.0776 × Protein% + 0.2534](1)

At the cheese factory gate, 1 kg of Grana Padano PDO cheese ripened 9 months without packaging was considered as the FU.

The scenario’s cheese yield (CY; kg of cheese/100 kg of milk in vat) was calculated according to the formula (Equation (2)) reported by Masotti et al. (2006) [32].

CY = [(Protein% × 76.6 × 0.944/100)/(65.5 − 5.8 − (0.655 × (((22.82 × Fat% × (1 − 0.14))/Protein% × 0.944/100))) + 22.56)] ×100(2)

### 2.5. System Boundaries and Allocation

**Figure 1:**
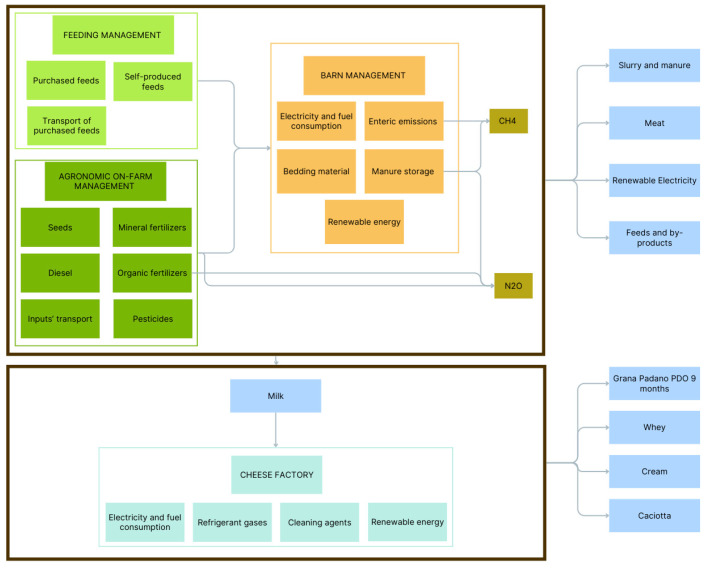
System boundaries considered for Life Cycle Assessment approach.

The environmental impact assessment was conducted according to the principles of a cradle-to-cheese factory gate approach, encompassing the lifecycle of the production process from agricultural input through to cheese manufacturing and ripening (Figure 1). The life stages of distribution, consumption, and disposal were excluded.

Specifically, the agricultural phase, encompassing the production of milk, feed (both self-produced and purchased feed), enteric manure emissions, and resource utilization such as energy, fuels, and bedding materials, received particular scrutiny. For the crop production stage, raw material production, manufacturing, and use were considered (i.e., fossil fuels, fertilizers, pesticides, seeds). Electricity, fossil fuels, and refrigerants consumed, with consideration also given to their production process, were taken into account for the cheese making and ripening stages. With regard to the cheese-making process, the amount of salt consumed was included, but lysozyme was excluded due to the low incidence and absence of data on the production process [33].

Milk production and cheese production are multifunctional processes with the potential to generate a range of products and by-products. According to ISO 14044 [28], the allocation of resources was avoided wherever possible. When necessary, the bio-physical allocation approach was employed, determining the allocation ratio (AF_milk_) between FPCM and meat at the farm level for sold calves and cows:

AF_milk_ = 1 − 5.7717 × BMR (3)

where BMR is the ratio between the mass of live weight of all animals sold per year and the mass of Fat and Protein Corrected Milk (FPCM) sold in the same period. This approach is based on the concept of energy use in relation to the physiological needs of the animal to produce milk and meat [31]. At the cheese factory level, milk was used for the production of Grana Padano PDO and other dairy products, including semi-skimmed milk, caciotta, and ricotta. By-products such as cream and whey were then obtained from the production of Grana Padano PDO. To assess the Grana Padano cheese impact, a weighted physical allocation considering the fat and protein content (GP) of the identified products was adopted by applying the following formula [31] (Table S1):

CF GP PDO (9 months) = Cheese Factory emissions (kg CO_2_eq/year) × [(FP_GP PDP 9 months × mass%_GP PDP 9 months)/(∑ FP_cheese coproducts × mass%_cheese coproducts) (4)

### 2.6. Life Cycle Inventory

Primary data were collected through previously drafted surveys administered by technicians on both farms and dairies. The study comprised 19 farms that sold all milk produced to a cheese factory located in the province of Piacenza (Italy). All the data refers to the year 2017.

### 2.7. Primary Data

The farms’ survey collected information related to milk and meat production, herd composition, feeding strategy, crop cultivation, manure management, and resource use (fuels, energy, bedding material). The primary data for this study was collected through direct interviews with farmers and was retrieved from farm management and documentation, including but not limited to agronomic effluent utilization plans, financial records, and farm notebooks.

The rations of all the animal categories were then analyzed with Razio-Best rationing software (v.560) to estimate the diets’ chemical and nutritional composition [34]. At the farm level, the data have been organized into four distinct clusters: feed production (FP), feed purchased (FA), resource use (RU), enteric emissions (EE), and manure emissions (ME).

Agronomic management focused exclusively on crops intended to be fed to livestock or used in anaerobic digesters. Specific data were collected on tillage practices for each individual crop cultivated, seed rates, annual production, type and rate of synthetic fertilizer, and pesticide application. Consumption data were also collected for sold crops to avoid allocation and discount inputs.

The cheese factory survey collected information on the amount of processed milk, milk transport, salt and detergents consumption, refrigerant consumption, and energy consumption related to the manufacturing process and ripening.

### 2.8. Emissions

The estimation of enteric methane emissions was conducted in accordance with the IPCC (2019) Tier 2 methodology [35] (Equation (5)):

Enteric CH_4_ = (GE × Ym)/55.65 (5)

where GE is the Gross Energy intake and Ym is the methane conversion factor. 

The emission factors (EF) were calculated for each animal category, including post-weaning calves, heifers, young cows, lactating cows, and dry cows. For pre-weaning calves, the EF was set to zero, as recommended by IPCC [35]. The calculations employed farm-specific data on diet digestibility (DE) and neutral detergent fiber (NDF) for each animal category, alongside parameters such as live weight, milk yield, and milk fat content.

Methane emissions from manure storage were estimated based on the manure volumes reported in the farm’s agronomic utilization plan and the application of Tier 2 equation no. 10.23 from IPCC [35] (Equation (6)). For each farm, Volatile Solids (VS) were calculated using IPCC equation no. 10.24, incorporating farm-specific dietary digestibility (DE) for each animal category, previously calculated Gross Energy (GE), and fixed values for Urinary Energy (UE; 0.04) and ash content (0.08), as recommended by IPCC [35] (Equation (7)). Methane conversion factor (MCF) values were drawn from IPCC tables, with consideration given to climate conditions, manure type (e.g., slurry, solid manure, digestate), and storage methods (e.g., uncovered, natural crust, covered). The maximum methane producing capacity (B_0_) was set at 0.24, as recommended by the IPCC [35].

Manure CH_4_ (kg) = vs. × (B_0_ × 0.67 × MCF) (6)

where VS is the Volatile Solid and MCF is the methane conversion factor

VS (kg) = GE × (1 − DE/100) + (UE × GE) × [(1 − ASH)/18.45](7)

where GE in the Gross Energy intake, UE is the urinary energy, and ASH is the diet ash content.

The nitrogen content of various manure types at the farm level and the adopted management practices were considered in the calculation of nitrous oxide (N_2_O) emissions, both direct and indirect, from manure storage. The calculations were conducted in accordance with Tier 1 equations no. 10.25, 10.28, and 10.29 from IPCC [35] (Equations (8) and (9)). The emission factor EF_3_ was defined based on the specific storage method and manure type for each farm for direct N_2_O emissions. The indirect N_2_O emissions (volatilization and leaching) were calculated with the following coefficients: EF_4_, set to 0.1 for all animal categories, and EF_5_ equal to 0.0075, Frac_GASms_ was set at 28% and 7% in the presence of an anaerobic biodigester, and Frac_LEACHms_ (leaching and run-off) was set at 0.1. The N_2_O emissions attributable to the use of synthetic nitrogen fertilizers were evaluated, with the emission factor (EF) employed being set at 0.022 kg N_2_O/kg N applied [36].

Direct Manure N_2_O (kg) = [[∑(∑ (Nex × AWMS) + N_cdg_] × EF_3_ ] × 44/28 (8)

where Nex is the nitrogen excretion by animals, AWMS is the manure management system, N_cdg_ is the annual nitrogen input via co-digestate, and EF_3_ is the emission factor for direct N_2_O emissions from manure management system

Indirect Manure N_2_O (kg) = [(N_vol_ × EF_4_) + (N_leach_ × EF_5_)] × 44/28 (9)

where N_vol_ is nitrogen volatilization, EF_4_ is the emission factor for N_2_O emissions from atmospheric deposition of nitrogen on soils and water surfaces, N_leach_ is the leaching nitrogen, and EF_5_ is the emission factor for N_2_O emissions from nitrogen leaching and runoff.

### 2.9. Secondary Data

Secondary data were derived from: Ecoinvent (version 3.8 and 3.9.1) [37], Agrifootprint (version 5) [38], and European Life Cycle Database (ELCD) (v.3.1; JRC-IES, 2015) [39].

### 2.10. Impact Assessment

The Life Cycle Inventory was analyzed using SimaPro software (version 9.5.0.0). Within the Life Cycle Impact Assessment (LCIA), the global warming potential (GWP 100 years) was assessed according to the IPCC (2021) methodology [40].

### 2.11. Statistical Analysis

Data were analyzed using R software (R Core Team, 2024; version 4.3.3; Vienna, Austria) [41]. The dataset was divided into three clusters (CL) based either on the tertiles of milk cheese yield (CY) variable or on the CF of 1 kg of FPCM. The normality of distributions was checked with Pearson (Nortest package, version 1.0.4). To test the existence of significant differences, a hypothesis test was performed using the Kruskal–Wallis rank sum test (package stats) followed by the post hoc Dunn’s test with the Bonferroni’s correction (package FSA). *p*-value ≤ 0.05 was considered statistically significant.

## 3. Results

### 3.1. Farm Description

The farms had an average of 167 ± 75 lactating cows with an average herd size of 298.52 ± 140.06 animal units (AU) and an average daily milk production of 28.55 ± 3.82 kg FPCM. The average land area was 94.64 ± 54.25 ha, mainly used for feed production (92.20 ± 15.24% of the total area), with a feed self-sufficiency of 66.47 ± 12.51% of the total dry matter consumed. The average stocking density was 3.51 ± 1.28 animal units (AU) per hectare. The coefficient for adult cows was set at 1, while that for cattle between six months and two years of age was set at 0.6, and that for cattle younger than six months was set at 0.4. Feed self-sufficiency was characterized by maize silage, ryegrass, and alfalfa hay, while purchased feeds were mainly concentrates and starch and protein meals.

The average carbon footprint of milk was 1.43 ± 0.30 kg CO_2_eq/kg FPCM. The milk CF value was mainly affected by enteric methane emissions (34%), feed purchasing (30%), manure emissions (24%), resource use (7%), and feed production (6%). Milk was delivered to a cheese-making plant, where it was processed into a variety of dairy products, including Grana Padano PDO cheese, skimmed milk, caciotta, and ricotta. The CF of cheesemaking materials was 78 ± 04 kg CO_2_eq/kg. The production process also resulted in the generation of cream and whey from the Grana Padano process making. The average CF of milk processed by the dairy was 1.38 kg CO_2_eq/kg FPCM, while the CF of Grana Padano PDO was 18.82 kg CO_2_eq/kg GP PDO 9 months. Meanwhile, the CF of skimmed milk was 2.27 kg CO_2_eq/kg, caciotta was 17.37 kg CO_2_eq/kg, cream was 0.82 kg CO_2_eq/kg, whey was 0.26 kg CO_2_eq/kg, and ricotta was 0.26 kg CO_2_eq/kg of product. Regarding GP production, the CF was mainly dependent on the agricultural phase (98.70% of CF), followed by energy and fuel consumption (1.14%) and other ingredients (0.13%). Data are reported in Table S2. Cheese factory inputs are shown in Table S3.

### 3.2. Cheese Yield Clusters

The results (mean ± standard error) are shown in Table 1. Cheese yield, expressed as kg GP per 100 kg of milk in the vat, was significantly higher in CL-CY3 (9.79 ± 0.08 kg) compared to CL-CY1 (9.36 ± 0.02 kg) and CL-CY2 (9.49 ± 0.02 kg), with a *p*-value of <0.001. Daily milk production (kg/d) differed significantly among the clusters, with CL-CY1 having the highest one (31.42 ± 0.78 kg), followed by CL-CY2 (28.86 ± 1.02 kg) and CL-CY3 (25.79 ± 2.17 kg) (*p* = 0.035).

Milk composition and FPCM production also showed significant differences across the groups. Fat content was highest in CL-CY3 (4.00% ± 0.03), significantly greater than CL-CY1 (3.80% ± 0.01) and CL-CY2 (3.83% ± 0.02) (*p* = 0.002). Similarly, protein content (%) was higher in CL-CY2 (3.48% ± 0.03) and CL-CY3 (3.50% ± 0.03) compared to CL-CY1 (3.38% ± 0.01) (*p* = 0.007). Daily FPCM production (kg/d) was significantly higher in CL-CY1 (30.86 ± 0.74 kg) than in CL-CY3 (26.13 ± 2.10 kg), with CL-CY2 (28.67 ± 1.03 kg) showing intermediate values (*p* = 0.039).

Regarding the carbon footprint, no significant differences were found in the CF of cheesemaking material, expressed as kg CO_2_eq/kg milk or kg CO_2_eq/kg. However, significant variation was observed for the CF of GP (9 months PDO). CL-CY1 exhibited the lowest CF (17.63 kg CO_2_eq/1 kg GP PDO), compared to CL-CY2 (20.03 kg CO_2_eq/1 kg GP PDO) and CL-CY3 (20.28 kg CO_2_eq/1 kg GP PDO) (*p* < 0.001).

Analysis of herd composition and management factors revealed no significant differences between the clusters in terms of the number of lactating cows, heifers, dry cows, or total herd size. Likewise, the stocking rate (AU/ha) and herd productivity (kg FPCM/AU) did not differ significantly across the groups.

In terms of feeding efficiency, no significant differences were found in herd-level feed use intensity (kg DM herd/kg FPCM) or lactating cows’ feed efficiency (FE; kg FPCM/kg DMI). Nevertheless, CL-CY1 showed the numerically highest feed efficiency index compared to CL-CY3. Enteric CH_4_ emissions were lower in CL-CY1 and higher in CL-CY3, with a tendency toward significance (*p* = 0.065).

Regarding purchased feed, significant differences were found in the use of milk powder for calves. CL-CY1 had the highest use (kg DM/kg FPCM) compared to CL-CY2 (*p* = 0.033). Furthermore, CL-CY2 had the highest level of self-produced non-leguminous hay (kg DM/kg FPCM) (*p* = 0.019). No significant differences were observed in the use of energy, fuels, or bedding materials across the clusters.

### 3.3. Milk Carbon Footprint Clusters

Results (mean ± standard error) are reported in Table 2. The carbon footprint of milk (1 kg FPCM) was found to be the lowest in CL-CF1 (1.14 kg CO_2_eq/kg FPCM), followed by CL-CF2 (1.40 kg CO_2_eq/kg FPCM), and highest in CL-CF3 (1.75 kg CO_2_eq/kg FPCM) (*p* < 0.001). The only discrepancy observed was in the contribution of the resource use cluster, which was lower in CL-CF1 and higher in CL-CF3. The former exhibited reduced LPG and purchased bedding materials consumption (*p* = 0.025 and *p* = 0.0014, respectively). Furthermore, a tendency towards a lower contribution of purchased feeds was observed in CL-CF1 (*p* = 0.065). It is noteworthy that CL-CF1 exhibited a complete absence of alfalfa hay purchases, while CL-CF3 demonstrated a higher level of such purchases (*p* = 0.048).

A similar pattern was observed in the CF of useful material in cheesemaking, protein, fat, and GP PDO (9 months), all of which also exhibited significant differences between the groups. The lowest CF was observed in CL-CF1 for useful material in cheesemaking (76.21 kg CO_2_eq/kg), protein (33.02 kg CO_2_eq/kg), and fat (29.33 kg CO_2_eq/kg). In contrast, CL-CF3 exhibited higher CF values of CF useful material (95.57 kg CO_2_eq/kg; *p* < 0.001). The CF for Grana Padano PDO cheese was found to be lowest in CL-CF1 (16.96 kg CO_2_eq/1 kg GP PDO), significantly lower than that of CL-CF3 (23.07 kg CO_2_eq/1 kg GP PDO) (*p* < 0.001).

No significant differences were detected in terms of milk yield, fat and protein milk content, and cheese yield (kg GP/100 kg milk in vat). Furthermore, no discrepancies were observed in terms of herd size and composition, stocking rate (AU/ha), and herd productivity (kg FPCM/AU). With regard to feeding strategies, CL-CF1 exhibited the lowest total heifer DMI, while CL-CF2 demonstrated the highest (*p* = 0.034). Conversely, CL-CF1 exhibited a higher dry cow ration digestibility (DE) (*p* = 0.027).

CL-CF1 exhibited the lowest nitrogen excretion rate (kg N/kg FPCM) and CH_4_ enteric emissions (kg CH_4_/kg FPCM), while CL-CF3 demonstrated the highest (*p* = 0.030). Furthermore, the nitrogen content in manure (kg N/kg FPCM) and N_2_O emissions (kg N2O/kg FPCM) from manure storage were lower in CL-CF1 than in CL-CF3 (*p* = 0.019 and *p* = 0.035, respectively).

## 4. Discussion

This study characterized the typical farms in the Piacenza (Italy) province for the production of Grana Padano PDO. Adherence to the product specification determines specific constraints with regard to the primary production stage, namely that for the animals’ feed, 75% of the fodder DMI must be from the specific production area. The cheesemaking process follows very specific procedures, even in the transformation phase [20]. In order to improve the carbon footprint of cheese, it is essential to enhance the primary production stage of milk production, which contributes most to the impact value of the final product [42].

In our findings the average milk CF, ranging from 0.95 to 2.14 kg CO_2_eq/kg FPCM, was in line with previous studies that analyzed the Italian scenario [23,24,43,44,45,46] but also consider the worldwide milk CF variation [5]. In particular, the results are consistent with those reported by Lovarelli et al. (2018), who considered dairy farms in the Grana Padano production system [23], but also with Froldi et al. (2022), analyzing the CF of raw milk produced in the Po Valley [26]. It is widely recognized that enteric emissions, manure emissions, and the purchase of externally sourced feed are the main contributors to impact values [47]. Overall, the variability in CF values is primarily influenced by animal welfare conditions, feeding strategies (e.g., silage inclusion level, feed and ration quality), the degree of feeds self-sufficiency, and the adoption of emission-reducing technologies during effluent storage (e.g., tank covers, anaerobic digestion plants) [48].

Considering Grana Padano cheese, the CF was slightly higher than that reported by Bava et al. (2018) (15.2 kg CO_2_eq/kg GP PDO 12 months) [24]. Gislon et al. (2023) showed a range from 10.60 to 12.20 kg CO_2_eq/kg GP PDO but the study applied an allocation factor at cheese factories based on products dry matter content [49]. Meanwhile, considering the latest proposals on environmental impact of GP, the Made Green in Italy Voluntary Scheme, based on screening studies, reports an average impact of 13.06 kg CO_2_eq/kg of product (considering a dry matter content of 68% at 9 months of ripening) [50]. Overall, the environmental impact of this Italian PDO product is consistent with those of other hard cheeses [51]. However, it is crucial to acknowledge that direct comparisons between results are not feasible due to the updating of IPCC Chapter 10 [40], the allocation factor, and the characterization methods employed [35].

**Figure 2:**
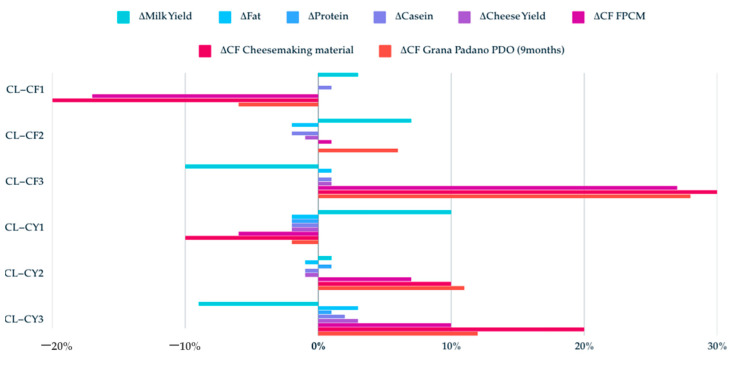
Evaluation of the effect of clusters (cluster based on milk cheese yield—CL-CY; cluster based on milk carbon footprint—CL-CF) on the variation from baseline of milk production and its titres, and of the carbon footprint (CF) of corrected milk production (FPCM), cheesemaking material, and Grana Padano PDO (9 months).

The application of cluster analysis permitted the evaluation of the effect of changes in milk CY or its CF on the CF of the cheese. This is of particular importance since the primary milk production phase represents the most significant contribution to the overall impact of cheese [51]. The results showed considerable variation in cheese yield and carbon footprint between clusters. This provides insights into potential improvement or deterioration scenarios under different management practices (Figure 2).

Cheese yield is the most important technological trait of milk and was found to be significantly higher in CL-CY3 (9.79 ± 0.08 kg) in comparison to CL-CY1 (9.36 ± 0.02 kg) and CL-CY2 (9.49 ± 0.02 kg) [52]. These findings indicate that despite the lower milk production (26.13 kg/d), CL-CY3 benefits from higher milk quality, particularly in terms of fat (4.00%), protein (3.50%), and casein (2.68%) content. These compositional advantages directly support cheese making efficiency [53,54]. On the other hand, a reduction in MY by 8% compared to the actual scenario led to a worsening of the carbon footprint performance, with a +10% increase in milk CF, a 20% increase in cheese-making useful material CF, and a +12% increase in GP. However, the opposite scenario, with an increase in MY of about 8%, led to a decrease in the CFs (−6% for FPCM, −10% for cheese-making useful material, −10% for GP). A clear trend towards a reduction in FPCM CF with an increase in yield has been widely demonstrated [5,15,47,55]. Improved production performance is a factor that is strongly related to genetic components and animal welfare conditions. In the context of the same production potential and higher welfare conditions, the feed efficiency (FE) of lactating cows is increased, thus reducing emissions per unit of product [8,55,56,57,58,59]. The CL-CY1 showed higher FE (1.34 kg FPCM/kg DMI) than other groups. This suggests a potential trade-off between achieving higher milk and FPCM yields with a reduced environmental footprint.

It is important to underline the importance of taking into account both the impact of the production level and the content of macronutrients such as fat and protein, which are essential from a nutritional and milk transformation point of view [60,61]. This was highlighted by FAO considering the application of nutritional Life Cycle Assessment approach (nLCA) [19]. To date, there have been no specific studies for the Italian scenario reporting on the impact on protein milk or cheese production. More frequently, the literature compares the effects of milk-based protein with those of plant-based drinks [62,63]. This approach is primarily employed for the purpose of guiding food choices; however, it could also be implemented to guide livestock farm improvement scenarios according to product destination. In the scenarios under consideration, the CF of cheesemaking material (protein plus fat) (CM) ranged from 51.05 to 117.32 kg CO_2_eq/kg CM. It was evident that variability was balanced between milk yield and quality.

The subsequent analysis of CF clusters indicated significant variations in the impact on milk and GP production. The CF for milk (1.14 kg CO_2_eq/kg FPCM) and GP PDO (16.96 kg CO_2_eq/kg GP) were at their lowest in CL-CF1. A 17% decrease in milk CF resulted in a 20% reduction in CF of cheesemaking material and a 6% reduction in CF of Grana Padano cheese. This outcome represents the optimal potential for enhancement. These performances were achieved with a moderate enhancement in productivity (3%, 29.42 kg/head/d), a lack of alteration in fat (3.89%) and protein (3.45%) content, and a one percentage point rise in casein content (2.66%). Conversely, a more substantial increase in milk production (+7%) but a reduction of up to 2% in fat and casein contents with the same protein showed a very modest (1%) worsening of CF for 1 kg FPCM but a 1% reduction for 1 kg cheese. Nevertheless, the increase in fat and casein (+1%) fails to compensate for a sharp reduction in production (−10%). These results align with Gislon et al. (2023) who show how the casein content but also the casein polymorphism affected the environmental performance of Grana Padano PDO cheese [49]. This finding suggests that genetic selection should be regarded as a strategy to mitigate environmental impact, rather than being exclusively focused on increasing production potential, with equal emphasis placed on the quality and type of casein [64,65,66]. In the Italian context, the ANAFIBJ proposal entails the incorporation of genetic selection indices for both Holstein and Jersey breeds, encompassing casein, feed efficiency, and nitrogen efficiency as integral components [67].

Compared to other clusters, the feeding strategy of CL-CF1 was based on the predominant use of forage silage and concentrates. This could indicate that a diet with higher digestibility supports a reduction in herd enteric emissions and nitrogen excretions per unit product (kg/kg FPCM). This was also in line with better FE and nitrogen use efficiency for lactating cows and a higher efficiency of nitrogen utilization at herd level (kg N/kg FPCM). The latter was then associated with a reduction in emissions from manure storage. It is widely acknowledged that enhancing nitrogen utilization is a pivotal metric of efficiency across both the animal and farm levels [68]. Numerous studies have documented that under conditions of enhanced efficiency, the resulting impacts are diminished [15,56,69,70]. Nevertheless, Rencricca et al. showed how different management systems, also in terms of crop management (annual or permanent meadows), could affect the farms’ environmental performances [21].

A potential shortcoming of the present study was that the farms under investigation did not differ in particular types of farming and crop management, as they were all located in the same province. However, the scenario analysis for enhancement or deterioration of CF metrics corroborates the mitigation object target.

## 5. Conclusions

This study provides a comprehensive characterization of typical dairy farms in the Piacenza Province in the Po Valley engaged in PDO Grana Padano production, with a particular focus on the key factors influencing environmental performance. The findings emphasize the pivotal role of high-quality feeding strategies in enhancing milk yield while concurrently reducing enteric methane emissions, nutrient excretion, and manure-related emissions. Improvements in milk yield, when coupled with adequate nutritional composition, have been observed to result in a decline in methane emissions per unit of milk and cheese. This finding underscores the strong correlation between productivity and environmental sustainability. The results of the study highlight a potential trade-off between maximizing milk yield and maintaining the quality of the milk, particularly in terms of fat and casein content, which are essential for cheese production. Despite its regional scope, the study provides valuable insights into CF mitigation strategies applicable to similar production systems. Future research should explore the impact of diverse crop and livestock management systems, as well as the potential of nutritional Life Cycle Assessment approaches, to refine sustainability strategies for dairy and cheese production.

## Figures and Tables

**Table 1:** The characterization of the clusters is based on milk cheese yield (CL-CY) with regard to the carbon footprint of milk, cheesemaking material, and Grana Padano PDO, as well as the inputs used by farms (milk production, feeding strategy, purchased food, agronomic management, energy consumption, and wastewater management). Data are reported as mean ± standard error.

Item	Unit	CL-CY1 ^1^ (n = 6)	CL-CY2 (n = 6)	CL-CY3 (n = 6)	*p*-Value
Carbon footprint:					
CF ^2^ milk	kg CO_2_eq/kg FPCM ^3^	1.30 ± 0.08	1.47 ± 0.04	1.52 ± 0.19	0.296
Feed purchased	kg CO_2_eq/kg FPCM	0.40 ± 0.04	0.45 ± 0.03	0.46 ± 0.10	0.581
Feed production	kg CO_2_eq/kg FPCM	0.07 ± 0.02	0.08 ± 0.01	0.08 ± 0.04	0.493
Resource use	kg CO_2_eq/kg FPCM	0.06 ± 0.01	0.10 ± 0.01	0.13 ± 0.03	0.064
Enteric emissions	kg CO_2_eq/kg FPCM	0.48 ± 0.02	0.48 ± 0.02	0.50 ± 0.07	0.960
Manure emissions	kg CO_2_eq/kg FPCM	0.29 ± 0.05	0.36 ± 0.05	0.36 ± 0.07	0.612
CF cheesemaking material	kg CO_2_eq/kg milk	0.09 ± 0.01	0.11 ± 0.003	0.12 ± 0.02	0.182
CF fat	kg CO_2_eq/kg milk	0.05 ± 0.003	0.06 ± 0.002	0.06 ± 0.01	0.220
CF protein	kg CO_2_eq/kg milk	0.04 ± 0.003	0.05 ± 0.001	0.05 ± 0.01	0.182
CF cheesemaking material	kg CO_2_eq/kg cheesemaking material	71.29 ± 4.62	79.94 ± 2.50	82.91 ± 10.40	0.296
CF fat	kg CO_2_eq/kg fat	33.55 ± 2.13	38.02 ± 1.07	38.61 ± 4.71	0.250
CF protein	kg CO_2_eq/kg protein	37.75 ± 2.49	41.91 ± 1.46	44.30 ± 5.69	0.347
CF GP	kg CO_2_eq/1 kg GP PDO ^4^ 9 months	17.63 ± 0.00 ^a^	20.03 ± 0.00 ^ab^	20.28 ± 0.00 ^b^	<0.001
Milk production:					
Milk production	kg/d	31.42 ± 0.78 ^a^	28.86 ± 1.02 ^ab^	25.79 ± 2.17 ^b^	0.035
FPCM production	kg/d	30.86 ± 0.74 ^a^	28.67 ± 1.03 ^ab^	26.13 ± 2.10 ^b^	0.039
Meat sold	t live weight/y	55.64 ± 14.43	29.76 ± 7.05	39.87 ± 12.00	0.414
AF ^5^ milk	%	86.02 ± 1.96	88.32 ± 1.53	85.67 ± 1.29	0.778
AF meat	%	13.98 ± 1.96	11.68 ± 1.53	14.33 ± 1.29	0.778
Cheesemaking material	%	7.19 ± 0.01 ^a^	7.31 ± 0.02 ^ab^	7.49 ± 0.06 ^b^	<0.001
Fat	%	3.80 ± 0.01 ^a^	3.83 ± 0.02 ^a^	4.00 ± 0.03 ^b^	0.002
Protein	%	3.38 ± 0.01 ^a^	3.48 ± 0.03 ^b^	3.50 ± 0.03 ^b^	0.007
Caseine	%	2.60 ± 0.02	2.62 ± 0.04	2.68 ± 0.04	0.421
Fat/caseine ratio	%	1.46 ± 0.01	1.46 ± 0.03	1.49 ± 0.02	0.281
Cheese Yield ^7^	kg GP/100 kg milk in vat	9.36 ± 0.02 ^a^	9.49 ± 0.02 ^ab^	9.79 ± 0.08 ^b^	<0.001
Cluster herd composition					
Lactating cow	n° AU ^6^	200.00 ± 31.87	139.67 ± 18.40	161.33 ± 37.72	0.341
Heifers	n° AU	120.13 ± 21.20	86.40 ± 17.16	104.03 ± 30.13	0.520
Dry cow	n° AU	34.33 ± 6.35	26.33 ± 3.66	23.33 ± 4.40	0.310
Herd size	n° AU	354.47 ± 58.76	252.40 ± 38.39	288.70 ± 71.54	0.359
Stocking rate	AU/ha	3.25 ± 0.30	3.65 ± 0.73	3.62 ± 0.53	0.612
Herd productivity	AU/kg FPCM	0.0002 ± 0.00001	0.0002 ± 0.00001	0.0002 ± 0.00003	0.097
CH_4_ enteric fermentation	kg/kg FPCM	0.02 ± 0.0005	0.02 ± 0.001	0.02 ± 0.002	0.065
Feeding strategy:					
DMI lactating cow	kg/d	23.14 ± 0.72	24.16 ± 1.29	22.29 ± 0.85	0.778
DMI heifers	kg/d	7.56 ± 0.64	7.74 ± 0.41	6.51 ± 0.50	0.140
DMI dry cow	kg/d	11.80 ± 0.67	8.33 ± 1.31	10.45 ± 0.64	0.058
Herd level feed use intensity	kg DMI herd/kg FPCM	1.03 ± 0.04	1.14 ± 0.04	1.18 ± 0.10	0.144
FE ^7^	kg FPCM/kg DMI	1.34 ± 0.04	1.20 ± 0.05	1.17 ± 0.09	0.144
DE ^8^ lactating cow	%DM	67.40 ± 1.15	63.97 ± 0.40	65.72 ± 1.70	0.081
DE heifers	%DM	60.79 ± 1.05	62.66 ± 0.85	62.00 ± 1.26	0.459
DE dry cow	%DM	60.32 ± 1.29	56.42 ± 1.89	58.88 ± 1.53	0.394
Nex ^9^ total herd	kg/kg FPCM	0.02 ± 0.001	0.02 ± 0.001	0.02 ± 0.002	0.994
N efficiency lactating cow	%	33.41 ± 1.93	30.36 ± 1.31	29.02 ± 2.13	0.414
Manure management:					
Slurry	m^3^/kg FPCM	0.002 ± 0.0003 ^a^	0.002 ± 0.0003 ^ab^	0.003 ± 0.0004 ^b^	0.028
Manure	m^3^/kg FPCM	0.001 ± 0.0003	0.002 ± 0.001	0.002 ± 0.001	0.523
N at field	kg y/ha	243.85 ± 20.63	262.71 ± 52.88	252.45 ± 42.05	0.884
N at field	kg/kg FPCM	0.012 ± 0.001	0.012 ± 0.0005	0.014 ± 0.002	0.587
CH_4_ storage emissions	kg/kg FPCM	0.005 ± 0.0005	0.004 ± 0.0002	0.005 ± 0.001	0.399
N_2_O storage and spreading emissions	kg/kg FPCM	0.0004 ± 0.00002	0.0004 ± 0.00002	0.0004 ± 0.0001	0.386
Energy and fuel consumption:					
Energy consumption	kWh/kg FPCM	0.04 ± 0.01	0.03 ± 0.01	0.05 ± 0.01	0.523
Renewable energy consumption	kWh/kg FPCM	0.003 ± 0.003	0.01 ± 0.005	0.06 ± 0.06	0.777
LPG ^10^	m^3^/kg FPCM	0.0005 ± 0.0002	0.002 ± 0.001	0.002 ± 0.002	0.243
Diesel consumed at farm	L/kg FPCM	0.01 ± 0.001	0.01 ± 0.002	0.02 ± 0.005	0.368
Diesel consumed to crop production	L y/ha	294.26 ± 12.77	289.37 ± 36.22	320.46 ± 26.68	0.581
Diesel consumed to crop production	L/kg FPCM	0.01 ± 0.002	0.02 ± 0.002	0.02 ± 0.01	0.927
Bedding materials	kg/kg FPCM	0.04 ± 0.01	0.13 ± 0.05	0.18 ± 0.08	0.343
Purchased feeds:					
Milk powder	kg DM/kg FPCM	0.001 ± 0.0004 ^a^	0.0001 ± 0.0001 ^b^	0.0004 ± 0.0003 ^ab^	0.033
Total hay	kg DM/kg FPCM	0.03 ± 0.02	0.08 ± 0.03	0.03 ± 0.02	0.412
Hay	kg DM/kg FPCM	0.02 ± 0.01	0.07 ± 0.03	0.02 ± 0.01	0.232
Alfalfa hay	kg DM/kg FPCM	0.01 ± 0.01	0.01 ± 0.01	0.02 ± 0.01	0.821
Starch meals	kg DM/kg FPCM	0.07 ± 0.02	0.11 ± 0.02	0.09 ± 0.04	0.390
Protein meals	kg DM/kg FPCM	0.05 ± 0.03	0.02 ± 0.02	0.02 ± 0.02	0.473
Concentrate	kg DM/kg FPCM	0.11 ± 0.05	0.19 ± 0.03	0.21 ± 0.03	0.325
Road transport	kg DM/km	121.19 ± 46.03	334.93 ± 158.92	114.65 ± 37.50	0.205
Agronomic management:					
Total land	ha	108.29 ± 15.76	96.50 ± 34.23	79.12 ± 12.72	0.364
Land self-feedproduction	% ha	96.71 ± 1.79	89.61 ± 8.75	90.28 ± 6.76	0.875
Land occupation	m^2^/kg FPCM y	0.50 ± 0.04	0.59 ± 0.14	0.85 ± 0.43	0.864
Feed self-sufficiency	% DM	72.18 ± 3.90	60.84 ± 4.15	66.41 ± 6.59	0.312
Total silage	kg DM/kg FPCM	0.46 ± 0.05	0.43 ± 0.06	0.68 ± 0.21	0.641
Corn silage	kg DM/kg FPCM	0.39 ± 0.04	0.36 ± 0.08	0.64 ± 0.23	0.738
Other silage	kg DM/kg FPCM	0.07 ± 0.05	0.07 ± 0.03	0.04 ± 0.03	0.825
Total hay	kg DM/kg FPCM	0.23 ± 0.05	0.26 ± 0.05	0.28 ± 0.15	0.587
Hay	kg DM/kg FPCM	0.02 ± 0.01 ^a^	0.11 ± 0.02 ^b^	0.04 ± 0.02 ^a^	0.019
Alfalfa hay	kg DM/kg FPCM	0.21 ± 0.05	0.15 ± 0.05	0.25 ± 0.15	0.520
N synthesis fertilization	kg/kg FPCM	0.01 ± 0.001	0.004 ± 0.001	0.01 ± 0.003	0.587
P synthesis fertilization	kg/kg FPCM	0.0001 ± 0.0001	0.00 ± 0.00	0.001 ± 0.001	0.364
Pesticides	g AI ^11^/kg FPCM	0.05 ± 0.01	0.05 ± 0.01	0.11 ± 0.03	0.242

^a.b.^ Different letters mean statistically significant differences *p* ≤ 0.05; ^1^ CY—yield of Grana Padano PDO cheese calculated by Masotti et al. (2006) equation [32]; ^2^ CF—carbon footprint; ^3^ FPCM—fat protein corrected milk; ^4^ GP PDO—Grana Padano Protected Denomination Origin cheese; ^5^ AF—allocation factor; ^6^ AU—Anima Unit; ^7^ FE—feed efficiency; ^8^ DE—digestible energy; ^9^ Nex—nitrogen excreted; ^10^ LPG—liquid propane gas; ^11^ AI—active ingredient.

**Table 2:** The characterization of the clusters is based on milk carbon footprint (CL-CF) with regard to the carbon footprint of milk, cheesemaking material, and Grana Padano PDO, as well as the inputs used by farms (milk production, feeding strategy, purchased food, agronomic management, energy consumption, and wastewater management). Data are reported as mean ± standard error.

Item	Unit	CL-CF1 (n = 6)	CL-CF2 (n = 6)	CL-CF3 (n = 6)	*p*-Value
Carbon footprint:					
CF ^1^ milk	kg CO_2_eq/kg FPCM ^2^	1.14 ± 0.05 ^a^	1.40 ± 0.02 ^ab^	1.75 ± 0.10 ^b^	<0.001
Feed purchased	kg CO_2_eq/kg FPCM	0.34 ± 0.08	0.41 ± 0.03	0.55 ± 0.04	0.065
Feed production	kg CO_2_eq/kg FPCM	0.05 ± 0.01	0.08 ± 0.01	0.11 ± 0.04	0.158
Resource use	kg CO_2_eq/kg FPCM	0.06 ± 0.01 ^a^	0.08 ± 0.01 ^ab^	0.15 ± 0.03 ^b^	0.032
Enteric emissions	kg CO_2_eq/kg FPCM	0.43 ± 0.04	0.50 ± 0.01	0.53 ± 0.05	0.242
Manure emissions	kg CO_2_eq/kg FPCM	0.26 ± 0.07	0.34 ± 0.02	0.41 ± 0.05	0.085
CF cheesemaking material	kg CO_2_eq/kg milk	0.08 ± 0.003 ^a^	0.10 ± 0.002 ^ab^	0.13 ± 0.01 ^b^	<0.001
CF fat	kg CO_2_eq/kg milk	0.04 ± 0.002 ^a^	0.05 ± 0.001 ^ab^	0.07 ± 0.01 ^b^	<0.001
CF protein	kg CO_2_eq/kg milk	0.04 ± 0.001 ^a^	0.05 ± 0.001 ^ab^	0.06 ± 0.004 ^b^	<0.001
CF cheesemaking material	kg CO_2_eq/kg cheesemaking material	62.35 ± 2.67 ^a^	76.21 ± 0.90 ^ab^	95.57 ± 5.57 ^b^	<0.001
CF fat	kg CO_2_eq/kg fat	29.33 ± 1.20 ^a^	36.10 ± 0.44 ^ab^	44.75 ± 2.34 ^b^	<0.001
CF protein	kg CO_2_eq/kg protein	33.02 ± 1.48 ^a^	40.11 ± 0.56 ^ab^	50.82 ± 3.24 ^b^	<0.001
CF GP	kg CO_2_eq/1 kg GP PDO ^3^ 9 months	16.96 ± 0.00 ^a^	19.12 ± 0.00 ^ab^	23.07 ± 0.00 ^b^	<0.001
Milk production:					
Milk production	kg/d	29.42 ± 1.25	30.78 ± 1.00	25.87 ± 2.09	0.082
FPCM production	kg/d	29.32 ± 1.10	30.45 ± 0.96	25.89 ± 1.97	0.104
Meat sold	t live weight/y	60.21 ± 16.44	37.66 ± 6.59	27.456 ± 6.66	0.312
AF ^4^ milk	%	85.19 ± 1.80	88.17 ± 1.63	86.65 ± 1.37	0.717
AF meat	%	14.81 ± 1.80	11.83 ± 1.63	13.35 ± 1.37	0.717
Cheesemaking material	%	7.34 ± 0.07	7.27 ± 0.04	7.39 ± 0.08	0.523
Fat	%	3.89 ± 0.04	3.82 ± 0.02	3.92 ± 0.05	0.342
Protein	%	3.45 ± 0.03	3.44 ± 0.03	3.46 ± 0.04	0.884
Caseine	%	2.66 ± 0.03	2.58 ± 0.03	2.67 ± 0.05	0.205
Fat/caseine ratio	%	1.46 ± 0.01	1.48 ± 0.02	1.47 ± 0.03	0.476
Cheese Yield ^5^	kg GP/100 kg milk in vat	9.56 ± 0.09	9.45 ± 0.04	9.64 ± 0.11	0.399
Cluster herd composition					
Lactating cow	n° AU ^6^	202.83 ± 42.43	165.33 ± 9.78	132.83 ± 27.91	0.392
Heifers	n° AU	125.80 ± 32.40	104.60 ± 13.94	80.17 ± 17.70	0.459
Dry cow	n° AU	31.00 ± 7.43	29.83 ± 2.24	23.17 ± 4.32	0.412
Herd size	n° AU	359.63 ± 80.79	299.77 ± 25.54	236.17 ± 49.11	0.366
Stocking rate	AU/ha	3.74 ± 0.31	3.23 ± 0.70	3.55 ± 0.56	0.587
Herd productivity	AU/kg FPCM	0.0002 ± 0.00001	0.0002 ± 0.00001	0.0002 ± 0.00003	0.172
CH_4_ enteric fermentation	kg/kg FPCM	0.02 ± 0.001 ^a^	0.02 ± 0.001 ^ab^	0.02 ± 0.002 ^b^	0.030
Feeding strategy:					
DMI lactating cow	kg/d	22.53 ± 0.61	25.01 ± 1.14	22.05 ± 0.76	0.097
DMI heifers	kg/d	6.39 ± 0.19 ^a^	8.15 ± 0.50 ^b^	7.27 ± 0.64 ^ab^	0.034
DMI dry cow	kg/d	11.00 ± 0.57	9.02 ± 1.61	10.56 ± 0.67	0.796
Herd level feed use intensity	kg DMI herd/kg FPCM	1.01 ± 0.04	1.12 ± 0.04	1.22 ± 0.10	0.135
FE ^7^	kg FPCM/kg DMI	1.31 ± 0.06	1.22 ± 0.05	1.17 ± 0.09	0.402
DE ^8^ lactating cow	%DM	65.55 ± 1.21	65.17 ± 1.19	66.37 ± 1.59	0.805
DE heifers	%DM	61.73 ± 1.32	61.40 ± 0.77	62.32 ± 1.16	0.751
DE dry cow	%DM	61.43 ± 1.21 ^a^	55.90 ± 1.71 ^b^	58.28 ± 1.29 ^ab^	0.027
Nex ^9^ total herd	kg/kg FPCM	0.02 ± 0.001 ^a^	0.02 ± 0.0004 ^ab^	0.02 ± 0.002 ^b^	0.030
N efficiency lactating cow	%	33.33 ± 1.94	30.60 ± 0.87	28.85 ± 2.34	0.421
Manure management:					
Slurry	m^3^/kg FPCM	0.002 ± 0.0004	0.002 ± 0.0003	0.003 ± 0.0004	0.166
Manure	m^3^/kg FPCM	0.002 ± 0.0004	0.001 ± 0.0005	0.003 ± 0.001	0.414
N at field	kg y/ha	259.46 ± 27.29	232.25 ± 49.87	267.30 ± 40.73	0.567
	kg/kg FPCM	0.01 ± 0.001 ^a^	0.01 ± 0.0004 ^ab^	0.01 ± 0.002 ^b^	0.019
CH_4_ storage emissions	kg/kg FPCM	0.004 ± 0.001	0.004 ± 0.0002	0.01 ± 0.0004	0.209
N_2_O storage and spreading emissions	kg/kg FPCM	0.0003 ± 0.00003 ^a^	0.0004 ± 0.00002 ^ab^	0.0005 ± 0.00004 ^b^	0.035
Energy and fuel consumption:					
Energy consumption	kWh/kg FPCM	0.04 ± 0.01	0.04 ± 0.01	0.04 ± 0.01	0.796
Renewable energy consumption	kWh/kg FPCM	0.06 ± 0.06	0.01 ± 0.01	0.00 ± 0.00	0.170
	% total energy consumption	17.88 ± 15.47	19.42 ± 9.29	0.00 ± 0.00	0.170
LPG ^10^	m^3^/kg FPCM	0.0001 ± 0.0001 ^a^	0.002 ± 0.001 ^b^	0.003 ± 0.001 ^ab^	0.025
Diesel consumed at farm	L/kg FPCM	0.01 ± 0.003	0.01 ± 0.002	0.01 ± 0.005	0.641
Diesel consumed to crop production	L y/ha	296.08 ± 19.84	284.24 ± 23.09	323.77 ± 34.75	0.717
Diesel consumed to crop production	L/kg FPCM	0.01 ± 0.002	0.02 ± 0.002	0.03 ± 0.01	0.278
Bedding materials	kg/kg FPCM	0.02 ± 0.01 ^a^	0.06 ± 0.01 ^ab^	0.26 ± 0.07 ^b^	0.0014
Purchased feeds:					
Milk powder	kg DM/kg FPCM	0.0006 ± 0.0003	0.0005 ± 0.0004	0.0008 ± 0.0005	0.751
Total hay	kg DM/kg FPCM	0.003 ± 0.003	0.05 ± 0.02	0.09 ± 0.03	0.080
Hay	kg DM/kg FPCM	0.003 ± 0.003	0.04 ± 0.02	0.06 ± 0.03	0.086
Alfalfa hay	kg DM/kg FPCM	0.00 ± 0.00 ^a^	0.01 ± 0.01 ^ab^	0.03 ± 0.01 ^b^	0.048
Starch meals	kg DM/kg FPCM	0.06 ± 0.03	0.10 ± 0.02	0.11 ± 0.03	0.310
Protein meals	kg DM/kg FPCM	0.002 ± 0.002	0.02 ± 0.02	0.06 ± 0.03	0.301
Concentrate	kg DM/kg FPCM	0.19 ± 0.05	0.18 ± 0.04	0.14 ± 0.04	0.796
Road transport	kg DM/km	117.72 ± 36.66	268.56 ± 173.50	184.49 ± 36.54	0.505
Agronomic management:					
Total land	ha	91.69 ± 17.38	117.96 ± 30.06	74.27 ± 16.40	0.554
Land self-feedproduction	% total land	97.35 ± 1.92	88.97 ± 8.61	90.28 ± 6.76	0.624
Land occupation	m^2^/kg FPCM y	0.44 ± 0.03	0.63 ± 0.13	0.87 ± 0.42	0.653
Feed self-sufficiency	% DM	71.93 ± 3.70	66.00 ± 3.12	61.49 ± 7.37	0.268
Total silage	kg DM/kg FPCM	0.50 ± 0.04	0.44 ± 0.06	0.63 ± 0.23	0.834
Corn silage	kg DM/kg FPCM	0.42 ± 0.05	0.39 ± 0.05	0.58 ± 0.24	0.960
Other silage	kg DM/kg FPCM	0.08 ± 0.05	0.05 ± 0.03	0.06 ± 0.04	0.937
Total hay	kg DM/kg FPCM	0.17 ± 0.04	0.28 ± 0.06	0.31 ± 0.14	0.459
Hay	kg DM/kg FPCM	0.04 ± 0.02	0.08 ± 0.03	0.05 ± 0.03	0.320
Alfalfa hay	kg DM/kg FPCM	0.13 ± 0.03	0.20 ± 0.06	0.26 ± 0.15	0.738
N synthesis fertilization	kg/kg FPCM	0.004 ± 0.001	0.005 ± 0.001	0.007 ± 0.003	0.612
P synthesis fertilization	kg/kg FPCM	0.001 ± 0.001	0.0001 ± 0.0001	0.00 ± 0.00	0.364
Pesticides	g AI ^11^/kg FPCM	0.07 ± 0.02	0.06 ± 0.01	0.08 ± 0.03	0.977

^a.b.^ Different letters mean statistically significant differences *p* ≤ 0.05; ^1^ CF: carbon footprint; ^2^ FPCM: fat protein corrected milk; ^3^ GP PDO—Grana Padano Protected Denomination Origin cheese; ^4^ AF—allocation factor; ^5^ CY—yield of Grana Padano PDO cheese calculated by the Masotti et al. (2006) equation [32]; ^6^ AU—Anima Unit; ^7^ FE—feed efficiency; ^8^ DE—digestible energy; ^9^ Nex—nitrogen excreted; ^10^ LPG—liquid propane gas; ^11^ AI—active ingredient.

## Data Availability

The original contributions presented in the study are included in the article/Supplementary Materials, further inquiries can be directed to the corresponding author.

## Supplementary Materials

The following supporting information can be downloaded at: https://www.mdpi.com/article/10.3390/ani15060811/s1, Table S1. Composition and allocation factors of dairy products derived from Grana Padano PDO cheese production; Table S2. Descriptive statistics of the farms evaluated; Table S3. Annual resource consumption and inputs for milk processing in the dairy plant.
