# Supplementary material for: Carbon Footprint Assessment of Dairy Milk and Grana Padano PDO Cheese and Improvement Scenarios: A Case Study in the Po Valley (Italy)
Source: Animals (Basel). 2025 Mar 12;15(6):811. doi: 10.3390/ani15060811 (PMC11939476; doi:10.3390/ani15060811)
Supplement: Supplementary file 1 [file animals-15-00811-s001.zip › animals-3519378-supplementary.pdf]

Supplementary materials

Table S1: Composition and allocation factors of dairy products derived from Grana Padano PDO cheese production.

	Fat	Protein	Fat and protein	Dry Matter	Production	Allocation Factor
	g/100g	g/100g	g/100g	%	t/y	
Whey	0.09	0.76	0.85	0.42	26130.00	12.58%
Cream	0.35	2.30	2.65	0.06	1456.00	2.19%
Skimmed milk	3.92	3.44	7.36	0.13	1280.00	5.34%
Caciotta	31.00	24.50	55.50	0.61	9.41	0.30%
Ricotta	10.90	8.80	19.70	0.24	11.89	0.13%
Grana Padano PDO (9 months)	28.00	33.00	61.00	0.68	2300.00	79.47%

Table S2. Descriptive statistic of the farms

Item	Unit	Mean	SD[1]	Min	Median	Max	CV[2]	Skewness	Kurtosis
Carbon footprint:									
CF[3] milk	kg CO_{2eq}/kg FPCM[4]	1.43	0.30	0.95	1.39	2.14	0.21	0.72	0.05
Feed purchased	kg CO_{2eq}/kg FPCM	0.43	0.15	0.05	0.46	0.69	0.35	-0.62	0.31
Feed production	kg CO_{2eq}/kg FPCM	0.08	0.06	0.01	0.07	0.29	0.80	1.96	4.45
Resource use	kg CO_{2eq}/kg FPCM	0.09	0.06	0.04	0.07	0.23	0.60	1.29	0.54
Enteric emissions	kg CO2eq/kg FPCM	0.49	0.10	0.25	0.48	0.77	0.20	0.56	3.16
Manure emissions	kg CO_{2eq}/kg FPCM	0.34	0.14	0.07	0.34	0.60	0.41	-0.10	-0.40
CF cheesemaking material	kg CO_{2eq}/kg milk	0.10	0.02	0.07	0.10	0.16	0.23	1.10	0.58
CF fat	kg CO_{2eq}/kg milk	0.06	0.01	0.04	0.05	0.09	0.24	1.18	0.78
CF protein	kg CO_{2eq}/kg milk	0.05	0.01	0.04	0.05	0.08	0.22	1.00	0.36
CF cheesemaking material	kg CO_{2eq}/kg cheesemaking material	78.04	16.29	51.05	76.11	117.32	0.21	0.70	0.06
CF fat	kg CO_{2eq}/kg fat	36.73	7.39	24.18	36.19	53.65	0.20	0.57	-0.19
CF protein	kg CO_{2eq}/kg protein	41.32	8.93	26.87	40.16	63.67	0.22	0.81	0.28
CF GP	kg CO_{2eq}/1 kg GP PDO[5] 9 months	19.31	1.23	17.63	20.03	20.28	0.06	-0.63	-1.66
Milk Production:									
Milk production	kg/d	28.69	4.10	16.63	29.59	32.87	0.14	-1.35	1.55
FPCM production	kg/d	28.55	3.82	16.96	29.58	32.30	0.13	-1.46	2.01
Meat sold	kg LW[6]/y	41,774.61	28,807.67	9,900.00	37,432.50	99,300.00	0.69	0.73	-0.66
AF[7] milk	%	86.67	3.91	78.90	86.39	94.87	0.05	-0.08	-0.47
AF meat	%	13.33	3.91	5.13	13.61	21.10	0.29	0.08	-0.47
Cheesemaking material	%	7.33	0.15	7.15	7.31	7.69	0.02	0.89	-0.13
Fat	%	3.88	0.10	3.74	3.84	4.09	0.03	0.82	-0.46
Protein	%	3.45	0.08	3.35	3.43	3.61	0.02	0.63	-0.84
Caseine	%	2.64	0.09	2.49	2.63	2.82	0.04	0.49	-0.65
Fat/caseine ratio	%	1.47	0.05	1.35	1.47	1.55	0.03	-0.34	-0.27
Cheese Yield[8]	kg GP/100 kg milk in vat	9.55	0.21	9.28	9.47	10.04	0.02	0.92	-0.32
Herd composition:									
Lactating cow	n° AU[9]	167.00	74.74	50.00	165.50	300.00	0.45	0.19	-1.06
Heifers	n° AU	103.52	55.82	30.00	101.10	228.60	0.54	0.42	-0.80
Dry cow	n° AU	28.00	12.32	8.00	25.50	55.00	0.44	0.27	-0.71
Herd size	n° AU	298.52	140.06	101.20	298.60	563.60	0.47	0.22	-1.12
Stocking rate	AU/ha	3.51	1.28	1.10	3.71	6.19	0.37	-0.15	-0.50
Herd productivity	AU/ kg FPCM	0.00	0.00	0.00	0.00	0.00	0.23	2.78	7.82
CH_4 enteric fermentation	kg/kg FPCM	0.02	0.00	0.02	0.02	0.03	0.14	2.59	7.18

Feeding strategy:									
DMI lactating cow	kg/d	23.20	2.40	19.60	23.19	30.23	0.10	1.00	1.81
DMI heifers	kg/d	7.27	1.33	5.11	6.87	9.82	0.18	0.46	-0.92
DMI dry cow	kg/d	10.19	2.59	3.15	10.93	13.18	0.25	-1.14	0.67
Herd level feed use	kg DMI herd y/AU	6,497.39	734.26	5,112.89	6,443.53	8,625.46	0.11	0.92	2.00
Herd level feed use intensity	kg DMI herd/kg FPCM	1.12	0.17	0.89	1.09	1.67	0.15	1.73	3.73
FE[10]	kg FPCM/kg DMI	1.23	0.16	0.87	1.26	1.49	0.13	-0.38	-0.51
DE[11] lactating cow	%DM	65.69	3.13	62.00	64.70	71.40	0.05	0.80	-0.93
DE heifers	%DM	61.82	2.58	56.20	61.83	65.95	0.04	-0.28	-0.75
DE dry cow	%DM	58.54	4.02	47.90	58.95	65.50	0.07	-0.90	0.85
Nex[12] total herd	kg/kg FPCM	0.02	0.00	0.01	0.02	0.03	0.17	1.59	3.91
N efficiency lactating cow	%	30.93	4.60	21.50	31.68	41.68	0.15	0.05	0.06
Manure management:									
Slurry	m^3/kg FPCM	0.002	0.001	0.001	0.002	0.004	0.44	0.34	-1.21
Manure	m^3/kg FPCM	0.002	0.001	0.000	0.002	0.006	0.76	0.98	0.99
N at field	kg y/ha	253.00	94.18	78.72	269.72	442.47	0.37	-0.15	-0.70
N at field	kg/ kg FPCM	0.01	0.00	0.01	0.01	0.02	0.24	2.35	6.08
CH_4 storage emissions	kg/ kg FPCM	0.00	0.00	0.00	0.00	0.01	0.32	-0.78	1.11
N_2O storage and spreading emissions	kg/ kg FPCM	0.00	0.00	0.00	0.00	0.00	0.23	0.83	1.56
Energy and fuel consumption:									
Energy consumption	kWh / kg FPCM	0.04	0.02	0.01	0.04	0.07	0.47	-0.03	-1.20
Renewable energy consumption	kWh/ kg FPCM	0.03	0.09	0.00	0.00	0.38	3.58	3.51	11.08
LPG[13]	m^3/ kg FPCM	0.00	0.00	0.00	0.00	0.01	1.56	2.20	4.59
Diesel consumed at farm	lt/ kg FPCM	0.01	0.01	0.01	0.01	0.04	0.55	1.57	2.01
Diesel consumed to crop production	lt y/ha	301.36	63.69	175.62	300.06	418.04	0.21	-0.13	-0.65
Diesel consumed to crop production	lt / kg FPCM	0.02	0.02	0.01	0.01	0.09	0.95	3.21	9.69
Bedding materials	kg/ kg FPCM	0.12	0.14	0.00	0.05	0.48	1.25	1.46	0.60
Purchased feeds:									
Milk powder	kg DM/kg FPCM	0.00	0.00	0.00	0.00	0.00	1.46	1.09	-0.41
Total hay	kg DM/kg FPCM	0.05	0.06	0.00	0.01	0.21	1.37	1.09	0.04
Hay	kg DM/kg FPCM	0.04	0.05	0.00	0.01	0.17	1.43	1.27	0.48
Alfalfa hay	kg DM/kg FPCM	0.01	0.02	0.00	0.00	0.06	1.85	1.29	-0.15
Starch meals	kg DM/kg FPCM	0.09	0.07	0.00	0.10	0.20	0.72	-0.12	-1.42

Protein meals	kg DM/kg FPCM	0.03	0.05	0.00	0.00	0.15	1.83	1.31	-0.11
Concentrate	kg DM/kg FPCM	0.17	0.10	0.00	0.18	0.35	0.59	-0.24	-1.01
Road transport	kg DM /km	190.26	248.76	9.52	120.19	1.120.61	1.31	2.83	7.93
Agronomic mamagement:									
Total land	ha	94.64	54.25	28.00	81.40	254.00	0.57	1.23	1.53
Land self-feedproduction	% total land	92.20	15.24	46.45	100.00	100.00	0.17	-1.96	2.67
Land occupation	m^2/kg FPCM y	0.65	0.62	0.30	0.44	2.98	0.96	3.01	8.43
Feed self-sufficiency	% DM	66.47	12.51	44.11	68.46	92.80	0.19	0.08	-0.48
Total silage	kg DM /kg FPCM	0.53	0.32	0.22	0.43	1.73	0.62	2.74	7.63
Corn silage	kg DM /kg FPCM	0.46	0.35	0.07	0.38	1.73	0.75	2.62	7.15
Other silage	kg DM /kg FPCM	0.06	0.09	0.00	0.00	0.28	1.38	1.00	-0.22
Total hay	kg DM /kg FPCM	0.25	0.22	0.04	0.23	1.00	0.87	1.97	4.17
Hay	kg DM /kg FPCM	0.06	0.06	0.00	0.03	0.19	1.13	0.89	-0.80
Alfalfa hay	kg DM /kg FPCM	0.20	0.23	0.00	0.15	1.00	1.14	2.34	5.70
N synthesis fertilization	kg/kg FPCM	0.01	0.00	0.00	0.01	0.02	0.74	2.49	6.59
P synthesis fertilization	kg/kg FPCM	0.00	0.00	0.00	0.00	0.01	3.76	3.52	11.10
Pesticides	g AI[14]/kg FPCM	0.07	0.05	0.01	0.07	0.24	0.73	1.70	3.35

[1] SD: Standard deviation; [2] CV: coefficient of variation; [3] CF: carbon footprint; [4] FPCM: fat protein corrected milk; [5]GP PDO: Grana Padano Protected Denomination Origin; [6] LW: live weight; [7]AF: allocation factor; [8] Cheese Yield calculated with Masotti et al. (2006) equation; [9] AU: animal unit; [10] FE: feed efficiency; [11] DE: digestible energy; [12] Nex: nitrogen excreted; [13]LPG: liquid propane gas; [14]AI: active substance.

Table S3. Annual resource consumption and inputs for milk processing in the dairy plant.

Inputs	um	
Milk road transport	km/y	76650
Input transport	km/y	4200
Diesel	l/ y	38325
Energy consumption	kW/y	1486136
Methane	m3/y	327550
R404A	kg/y	10
R407C	kg/y	12
R407F	kg/y	130
Salt	kg/y	10800
Sodium carbonate	kg/y	14025
Acid cleaners	kg/y	12375
